# A learnable EEG channel selection method for MI-BCI using efficient channel attention

**DOI:** 10.3389/fnins.2023.1276067

**Published:** 2023-10-20

**Authors:** Lina Tong, Yihui Qian, Liang Peng, Chen Wang, Zeng-Guang Hou

**Affiliations:** ^1^China University of Mining and Technology-Beijing, Beijing, China; ^2^State Key Laboratory of Multimodal Artificial Intelligence Systems, Institute of Automation, Chinese Academy of Sciences, Beijing, China; ^3^Chinese Academy of Sciences (CAS) Center for Excellence in Brain Science and Intelligence Technology, Beijing, China

**Keywords:** brain-computer interface, motor imagery, channel selection, deep learning, attention mechanism

## Abstract

**Introduction:**

During electroencephalography (EEG)-based motor imagery-brain-computer interfaces (MI-BCIs) task, a large number of electrodes are commonly used, and consume much computational resources. Therefore, channel selection is crucial while ensuring classification accuracy.

**Methods:**

This paper proposes a channel selection method by integrating the efficient channel attention (ECA) module with a convolutional neural network (CNN). During model training process, the ECA module automatically assigns the channel weights by evaluating the relative importance for BCI classification accuracy of every channel. Then a ranking of EEG channel importance can be established so as to select an appropriate number of channels to form a channel subset from the ranking. In this paper, the ECA module is embedded into a commonly used network for MI, and comparative experiments are conducted on the BCI Competition IV dataset 2a.

**Results and discussion:**

The proposed method achieved an average accuracy of 75.76% with all 22 channels and 69.52% with eight channels in a four-class classification task, outperforming other state-of-the-art EEG channel selection methods. The result demonstrates that the proposed method provides an effective channel selection approach for EEG-based MI-BCI.

## 1. Introduction

A brain-computer Interface (BCI) provides an interface between users and external devices by converting brain signals into commands (Fadel et al., 2020). Electroencephalography (EEG), magnetoencephalography (MEG), functional magnetic resonance imaging (fMRI), and functional near-infrared spectroscopy (fNIRS), et al., are commonly used to acquire signals from the brain (Herrera-Vega et al., 2017; Berger et al., 2019; Saha et al., 2021). Among these techniques, EEG is currently one of the most popular brain-imaging techniques for its non-invasive nature, portability, and low cost (Abiri et al., 2019; Aggarwal and Chugh, 2022). EEG-based BCI can be applied to various tasks, such as seizure detection, workload, motor imagery (MI), and emotion recognition (Das Chakladar et al., 2020; Lashgari et al., 2020; Gu et al., 2021). In the MI task, subjects are asked to imagine human movements without performing them. EEG signals generated during this process are collected, and the intention can thus be recognized (Chaisaen et al., 2020; Khan et al., 2020). MI-BCI has numerous applications for aiding the elderly and disabled (Lazarou et al., 2018; Palumbo et al., 2021; Saibene et al., 2023).

MI-BCI is built on the fact that the brain evokes event-related synchronization (ERS) and event-related desynchronization (ERD) at different locations over the scalp when imagining body movements (Lu et al., 2017; Zhang et al., 2021). Researchers have progressively placed more and more electrodes on the subject's scalp to record signals for more detailed information, making channel selection a critical stage for EEG-based BCI (Abdullah et al., 2022). Channel selection aims to identify a small subset of channels that involves the most classified information. It can reduce computational costs and the interference of irrelevant EEG channels. Thus, the most important issues in channel selection are: how many channels are suitable, and which channels should be selected. This work focuses on the number of channels, that is ensuring the high recognition accuracy while minimizing the channel subset.

Researchers have been making many effort to find a suitable channel selection method. These methods can be generally classified as filtering, wrapper, embedded, and hybrid techniques. Utilizing wrapper techniques for channel selection demands a substantial computational resource investment. Tang et al. (2022) introduced the sequential backward floating search (SBFS) method into EEG channel selection. While significant improvements have been made to reduce training time, it still necessitates ~2,000 seconds or more to complete channel selection. Therefore, this paper focuses on filtering and embedded techniques. The filtering technique is independent of subjects and classifiers, high-speed, stable, and does not consume significant computational resources (Baig et al., 2020), and the embedded technique can be used with deep learning techniques. Most filtering techniques are performed based on the statistical information of the EEG signal. Tam et al. (2011) proposed a channel selection method based on common spatial pattern (CSP) filter coefficient ranking for MI classification, called CSP-rank. They conducted a 20-session experiment with 64-channel EEG from five chronic stroke patients and found that the average classification accuracy of CSP-rank for 8–38 electrodes remained above 90%. Twenty-two electrodes achieved the highest average accuracy of 91.70%. Arvaneh et al. (2011) adopted a new filtering approach with a pre-specified subset channel selection scheme. They proposed a sparse common spatial pattern (SCSP) algorithm instead of CSP for optimal EEG channel selection. The results show that the SCSP algorithm outperformed the existing algorithms, including Fisher discriminant, mutual information, support vector machine and CSP. If the goal is to minimize the number of channels while maintaining comparable average accuracy to using all channels, SCSP achieved 79.07% accuracy with an average of 8.55 channels on the first dataset and 79.28% accuracy with an average of 7.6 channels on the second dataset. Shi et al. (2023) proposed an EEG channel selection method based on sparse logistic regression (SLR). This method was compared to conventional channel selection based on correlation coefficients (CCS) using a 64-channel two-class MI dataset. In the scenarios of selecting 10 channels and 16 channels, the accuracy achieved by the proposed method were 86.63 and 87.00%, respectively, demonstrating a performance advantage of 4.33 and 2.94% over CCS.

In recent years, deep learning techniques have shown advantages in big data processing. Several attempts have been made to explore an optimal embeded channel selection method. Zhang et al. (2021) proposed a deep learning-based approach to automatically select the relevant EEG channels while recognizing two MI states. A sparse squeeze-and-excitation (SE) module is used to learn EEG channels' contribution to MI classification, which developed into an automatic channel selection strategy. The results show that this method indicates a 3.30% improvement compared to CSP in their private dataset. Strypsteen and Bertrand (2021) employed a concrete selector layer to optimize both the channel selection and the network weights in an end-to-end manner. This layer uses a Gumbel-softmax method to deal with the discrete parameters inherent to a subset selection problem. It can freely specify the number of channel subsets by modifying the loss function so that the network does not pick the same channels as much as possible. Their method was evaluated on two EEG tasks: motor execution and auditory attention decoding. The Gumbel-softmax performs at least as well as (often better than) state-of-the-art methods: mutual information for motor execution and greedy channel selection with the utility metric for auditory attention decoding. These studies show that using deep learning techniques instead of traditional signal processing methods is credible and can substantially improve performance.

This paper proposes a new method for EEG channel selection by introducing efficient channel attention (ECA) modules into a convolutional neural network (CNN) (Wang et al., 2020). An ECA module can recalibrate the channels based on feature interdependencies, allowing the network to learn each EEG channel's importance to classification for each subject and improve performance. Using the proposed approach, a personalized optimal channel subset can be obtained based on the order of channels in the learned channel importance ranking. This ensures that the channel subset includes the most discriminative channels for each subject. The main contributions of this work are as follows:

1. An innovative method is proposed to find an optimal channel subset for each subject with the ECA module. The subset is formed based on the importance of each channel for that subject in the MI classification process. Researchers can easily adjust the number of channels according to actual needs and hardware conditions.
2. ECA modules are added between the convolutional layers of the CNN to recalibrate feature interdependencies between channels adaptively. A CNN structure called ECA-DeepNet based on DeepNet (Schirrmeister et al., 2017) is proposed, and classification accuracy of the network improves with minimal computational cost.

The rest of the paper is organized as follows. Section 2 describes the detail of the ECA module and the proposed method for channel selection. Section 3 presents the experimental results to validate and compare the method with state-of-the-art methods. Some discussions are given in Section 4.

## 2. Materials and methods

**Figure 1:**
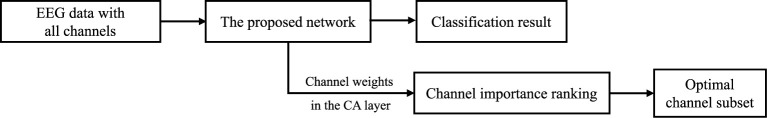
Overview of the proposed channel selection method.

This section presents a detailed description of the proposed channel selection method. As depicted in Figure 1, the entire process consists of four main steps: (1) training the proposed model with the data from the subject, (2) extracting the channel weights from the channel attention (CA) layer of the trained network, (3) ranking the importance of each channel based on the extracted weights, and (4) selecting a certain number of channels from the ranking to form an optimal channel subset for that subject.

### 2.1. Dataset and data preprocessing

To analyze the EEG signals and evaluate the proposed method, the publicly available MI-EEG dataset “BCI Competition IV dataset 2a (BCIC IV 2a dataset)” is introduced (Brunner et al., 2008). This dataset comprises four different MI tasks: the movement of the left hand, right hand, feet, and tongue. Each task lasts for 4 s from cue onset to the end of the task, and no feedback is provided. The dataset contains 22-channel EEG data from nine subjects, and each subject has two sets of data, namely the training set and testing set. Each set includes 288 trials of MI data, with 72 trials for each of the four tasks. The EEG data were initially sampled at a frequency of 250 Hz and filtered with a bandpass filter between 0.5 and 100 Hz, as well as a notch filter at 50 Hz.

The EEG signals were preprocessed as follows. A bandpass filter between 1 and 40 Hz was applied to reduce the effect of eye blinking and extract the information related to MI. An exponential moving average with a decay factor of 0.999 was applied to each channel to normalize the continuous data. The data was then segmented into four-second time windows with a sliding window over the time period from −0.5 to 4 s for each trial. After cropped by the sliding window, there are 864 samples for each subject in the training set and testing set separately, with 216 samples for each of the four tasks. Each sample consists of MI data from 22 channels, and each channel contains 1,000 sampling points.

### 2.2. Channel attention layer

To perform channel selection, evaluating the correlation between channels is necessary. Irrelevant channels are deemed redundant and not included in the channel subset. The ECA module is a channel attention module based on the attention mechanism (Wang et al., 2020). It can learn the interdependencies among different feature maps from the channel dimension and adaptively adjust the channel features by assigning weights to each channel. By placing the ECA module as the first layer of the network, it can learn the interdependence among EEG channels. Specifically, it scores the importance of each EEG channel. It assigns weights to strengthen the crucial channels while weakening the influence of less important channels on the rest of the model. This score also serves as the basis for subsequent channel selection. As a result, this layer is referred to as the channel attention (CA) layer here. The ECA module's efficiency lies on avoiding channel dimensionality reduction and enabling appropriate local cross-channel interactions. Next, the working mechanism of the ECA module will be explained, illustrating on how it achieves these two objectives.

The input of the ECA module is denoted as *X*∈ℝ^*W*×*H*×*C*^, where *W*, *H* and *C* are width, height and channel dimension. When the ECA module serves as the CA layer, the input is the EEG data, and at this point, *W* = 1. The ECA module first calculates the aggregated features *y*∈ℝ^1 × 1 × *C*^ along the channel dimension without dimensionality reduction, which is represented as


(1) $$\begin{array}{l} y=g\left(X\right)=\frac{1}{WH}\overset{W,H}{\underset{i=1,j=1}{\sum }}X_{i,j}, \end{array}$$

where *g*(*X*) is channel-wise global average pooling (GAP). This operation aggregates feature maps of the EEG sample across time-space dimensions *W*×*H*, producing channel-wise statics embedded with the global distribution of feature responses. In general, channel attention can be learned by


(2) $$\begin{array}{l} w=\sigma \left(\text{W}y\right), \end{array}$$

where **W** involves *C*×*C* parameters, and σ is a Sigmoid function. However, the ECA module employs a band matrix **W_k_** to learn channel attention:


(3) $$\begin{array}{l} \left[\begin{array}{cccccccc} w^{1,1} & \cdots & w^{1,k} & 0 & 0 & \cdots & \cdots & 0\\ 0 & w^{2,2} & \cdots & w^{2,k} & 0 & \cdots & \cdots & 0\\ \vdots & \vdots & \vdots & \vdots & \ddots & \vdots & \vdots & \vdots \\ 0 & \cdots & 0 & 0 & \cdots & w^{C,C-k+1} & \cdots & w^{C,C} \end{array}\right], \end{array}$$

where **W_k_** is a *k*×*C* parameter matrix with much fewer parameters than **W**. As in Equation (3), the weight of *y*_*i*_ only considers the interaction between *y*_*i*_ and its adjacent *k* neighbors, and it will be more efficient to make all the channels share the same learning parameters, i.e.,


(4) $$\begin{array}{l} w_{i}=\sigma \left(\overset{k}{\underset{j=1}{\sum }}w^{i}y_{j}^{i}\right),y_{j}^{i}\in \Omega_{i}^{k}, \end{array}$$

where $\Omega_{i}^{k}$ indicates the set of *k* adjacent channels of *y*_*i*_. Such a strategy can be easily implemented by fast 1*D* convolution with a kernel size of *k*, which can be described as


(5) $$\begin{array}{l} w=\sigma \left({\text{Conv1d}}_{k}\left(y\right)\right), \end{array}$$

where Conv1d is 1*D* convolution.

Since the ECA module aims to capture local cross-channel interaction appropriately, the kernel size *k* of 1*D* convolution must vary with different channel dimensions *C*. High channel dimension needs longer range interaction, and low channel dimension has shorter range interaction. Therefore, the kernel size *k* should be adaptively determined by channel dimension *C*, i.e.,


(6) $$\begin{array}{l} k=\psi (C)={\left|\frac{log_{2}(C)}{2}+\frac{1}{2}\right|}_{odd}, \end{array}$$

where |*k*|_*odd*_ indicates the nearest odd number of *k*.

However, when the ECA module is used as the CA layer, it is required to no longer focus on local, but capture global cross-channel interactions in order to learn feature interdependencies between EEG channels thoroughly. This is because EEG channels interact between both adjacent and non-adjacent channels, especially within corresponding regions of the contralateral brain hemisphere. Therefore, the 1*D* fast convolution in the CA layer is replaced by a fully connected layer. The number of neurons in this fully connected layer matches the channel dimension *C*, thus avoiding the influence of dimension reduction on channel attention learning, i.e.,


(7) $$w_{CA}=\sigma (W_{CA}y+b),$$

where **W_CA_** represents the weight parameter matrix of the fully connected layer, and *b* is the bias coefficients. This fully connected layer will incur additional computational costs, but it ensures that the CA layer considers all channels when learning the importance of each channel. Then channel weights with the same length as channel dimension *C* are obtained, and the ECA module will output the recalibrated feature maps along the channel dimension without altering the dimensions of the input samples, i.e.,


(8) $$\begin{array}{l} Y=w_{\left(CA\right)}\cdot X,Y\in {\mathbb{R}}^{W\times H\times C} \end{array}$$

**Figure 2:**
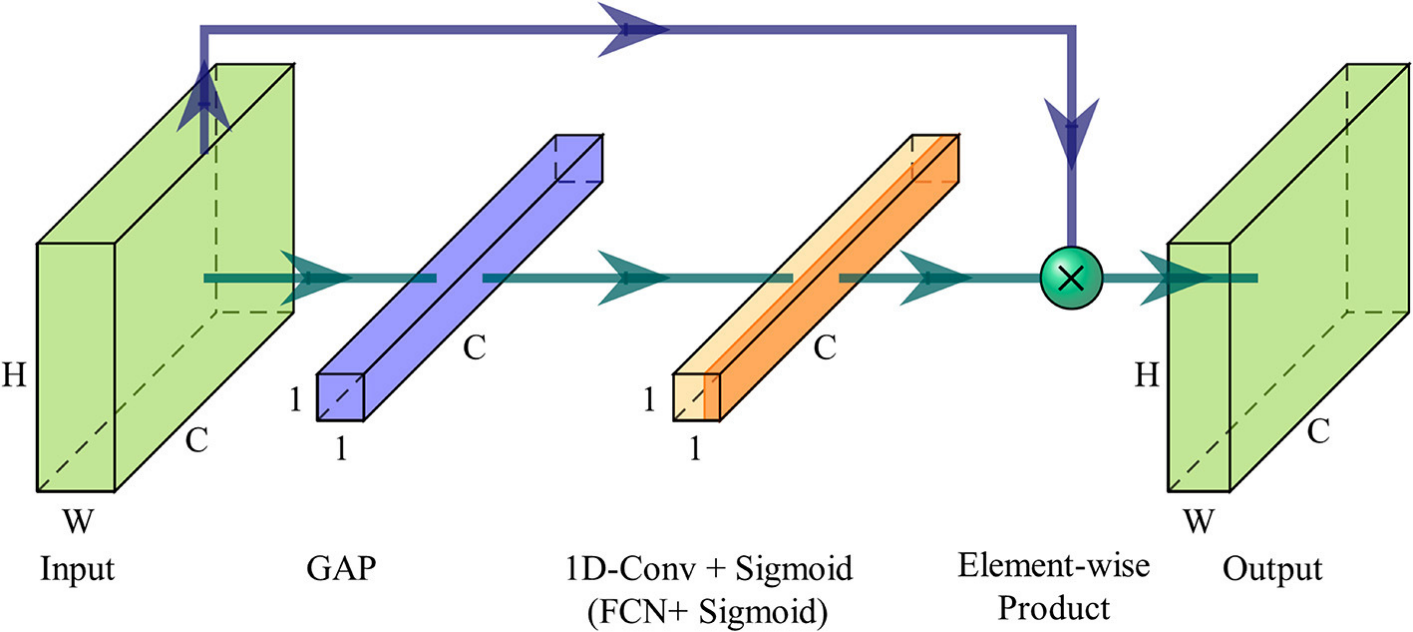
Diagram of an ECA module. When the ECA module is utilized as the CA layer, 1*D* fast convolution is substituted with a fully connected layer.

Figure 2 illustrates the overview of an ECA module. After aggregating feature maps using GAP, the ECA module uses a 1*D* fast convolution with adaptive kernel size *k*, followed by a Sigmoid function to learn the channel attention. Finally, input features are recalibrated from the channel dimension by assigning weights.

### 2.3. ECA modules in DeepNet

**Figure 3:**
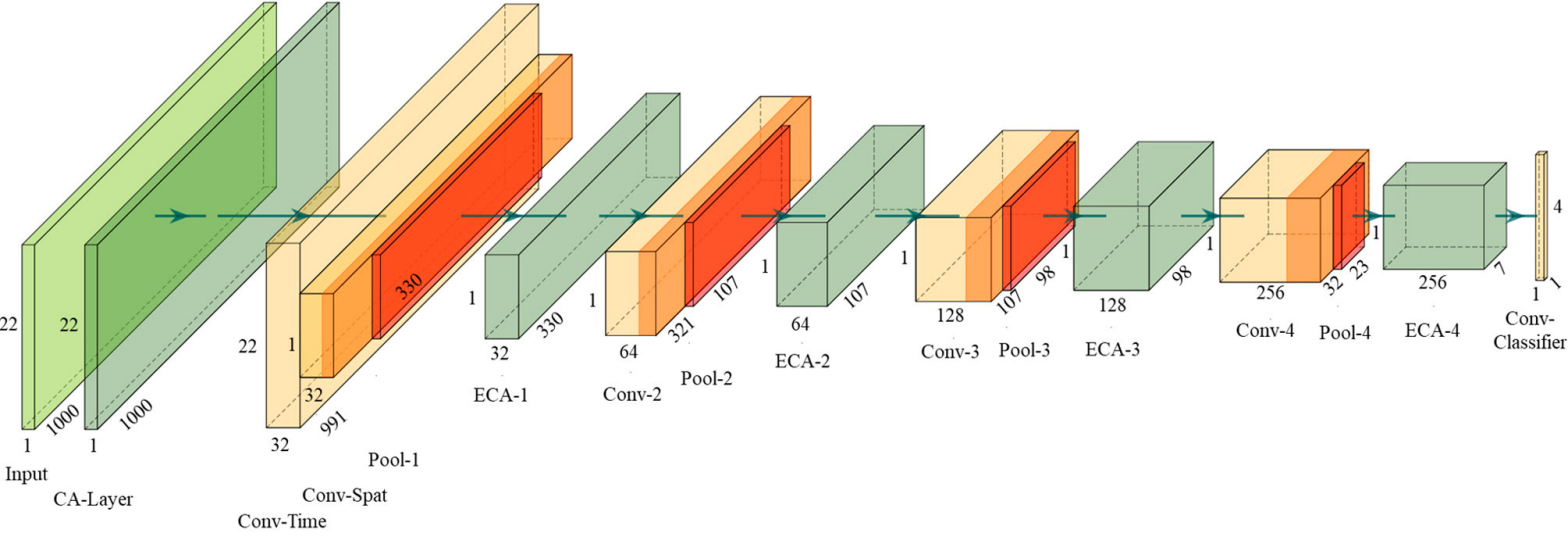
Proposed ECA-DeepNet architecture. The dimension of all layers are depicted as well.

In addition to placing the ECA module as the CA layer to learn the importance of EEG channels, positioning the ECA module between the convolutional layers of the network also serves to highlight significant feature maps and suppress irrelevant ones, thereby improving classification performance. To demonstrate the effects, this paper integrates the ECA modules into DeepNet (Schirrmeister et al., 2017), which is one of the most highly cited open-source models (Dai et al., 2020). It splits the first convolutional layer into a first convolution across time and a second convolution across space (electrodes), exploiting the ERS and ERD phenomena more effectively. Four ECA modules are added between convolution layers, and an additional ECA module is set before the first layer to act as the CA layer. Termed as ECA-DeepNet, the complete architecture is depicted in Figure 3.

Exponential linear units (ELUs) (Clevert et al., 2016), denoted as Equation (9), are selected as activation functions since they can speed up the learning process and improve classification accuracy.


(9) $$ELU(x)=\{\begin{array}{ll} x, & \text{if }x>0,\\ e^{x}-1, & \text{otherwise}. \end{array}.$$

The detailed parameters of the proposed ECA-DeepNet architecture are given in Table 1. Note that the number of filters in four convolution-max-pooling blocks are changed from (25, 50, 100, 200) to (32, 64, 128, 256) to fit the mapping as shown in Equation (6) better.

**Table 1:** Architecture of the proposed ECA-DeepNet.

**Block**	**Layer**	**Filters**	**Size of kernel**	**Output**	**Activation**	**Parameters**
	Input			22 × 1,000		
	Reshape			22 × 1,000 × 1		
	CA-Layer			22 × 1,000 × 1		506
	Reshape			1 × 1,000 × 22		
1	Conv-Time	32	10 × 1	32 × 991 × 22	Linear	320
	Conv-Spat	32	1 × 22	32 × 991 × 1	Linear	22,528
	BatchNorm-1			32 × 991 × 1		64
	Activation-1			32 × 991 × 1	ELU	
	Pool-1		3 × 1	32 × 330 × 1		
	ECA-1			32 × 330 × 1		4
	dropout-1			32 × 330 × 1		
2	Conv-2	64	10 × 1	64 × 321 × 1	Linear	20,480
	BatchNorm-2			64 × 321 × 1		128
	Activation-2			64 × 321 × 1	ELU	
	Pool-2		3 × 1	64 × 107 × 1		
	ECA-2			64 × 107 × 1		4
	dropout-2			64 × 107 × 1		
3	Conv-3	128	10 × 1	128 × 98 × 1	Linear	81,920
	BatchNorm-3			128 × 98 × 1		256
	Activation-3			128 × 98 × 1	ELU	
	Pool-3		3 × 1	128 × 32 × 1		
	ECA-3			128 × 32 × 1		6
	dropout-3			128 × 32 × 1		
4	Conv-4	256	10 × 1	256 × 23 × 1	Linear	327,680
	BatchNorm-4			256 × 23 × 1		512
	Activation-4			256 × 23 × 1	ELU	
	Pool-4		3 × 1	256 × 7 × 1		
	ECA-4			256 × 7 × 1		6
	Conv-Classifier	4	7 × 1	4 × 1 × 1	Linear	7,172
	Activation-4			4 × 1 × 1	Logsoftmax	
	Reshape			4		

### 2.4. Proposed channel selection method

The channel selection method proposed in this paper is based on the weights within the CA layer of the network. As described in Section 2.2, during the network training on a subject's EEG data, the CA layer automatically learns the attention between channels, assigning corresponding weights based on the importance of each EEG channel in the classification task. More important channels are assigned higher weights, while less important channels are assigned lower weights. Therefore, ECA-DeepNet can be used to train a model for each subject on BCIC IV 2a dataset. The channel weights *w* in the CA layer of each model can be collected after the training process.

After reordering channels by the extracted weights, the importance ranking of EEG channels for each subject can be described as


(10) $$\begin{array}{l} R_{i}=\left[ch_{1},ch_{2},...,ch_{C}\right],w_{ch_{1}}>w_{ch_{2}}>...>w_{ch_{C}}, \end{array}$$

where *ch*_*j*_ is the channel name with the j-th largest *w*_*ch*_. Finally, the optimal channel subset for subject *i* can be obtained from the ranking *R*_*i*_, i.e.,


(11) $$\begin{array}{l} S_{i}=R_{i}\left[1:N_{c}\right]=\left[ch_{1},ch_{2},...,ch_{N_{c}}\right], \end{array}$$

where *N*_*c*_ is the number of channels in the channel subset. Researchers can freely determine the size of the channel subset *N*_*c*_ according to their specific needs.

## 3. Results

### 3.1. Hyperparameter optimization

Structural hyperparameters of the ECA-DeepNet architecture have been specified and presented in Table 1. For the hyperparameter optimization, the open-source framework Optuna was employed in this paper (Akiba et al., 2019). This framework uses the Tree-structured Parzen Estimator (TPE) to progressively reduce the parameter search space until it finds the optimal value. The hyperparameter search space in the network is shown in Table 2.

**Table 2:** Search space for hyperparameters.

**Name**	**Range**	**Type**
Dropout rate	(0, 0.9)	Discrete (0.1)
Optimizer	AdamW, Adadelta, and Adagrad	Choice
Learning rate	(10^−5^, 10^−1^)	Continuous (exponential distribution)
Batch size	(4, 8, 16, 32, 64)	Choice
Weight decay	(10^−5^, 10^−4^, 10^−3^, 10^−2^)	Choice

The content in brackets in “Type” column is supplementary to the details, “Discrete (0.1)” indicates a discrete variable with a step size of 0.1 and “Continuous (exponential distribution)" indicates that the continuous variable follows an exponential distribution.

This study aims to find the optimal subject-specific classification model. Therefore, each subject obtained a corresponding set of best hyperparameters with and without channel selection during the hyperparameter search. When searching, the training set of BCIC IV 2a dataset was re-split into a training set and a validation set in a radio of 8:2. The best-performing set of hyperparameters was determined based on the highest accuracy on the validation set. Additionally, experiments took place on NVIDIA TITAN V GPUs. To ensure the replicability of results across all experiments, a consistent random seed of 20200220 was employed. Table 3 represents the results of best hyperparameters tuning by Optuna for each subject in BCIC IV 2a dataset.

**Table 3:** Hyperparameters for each subject in BCIC IV 2a dataset.

**Subject**	**Hyperparameters before/after channel selection**				

	**Dropout rate**	**Optimizer**	**Learning rate**	**Batch size**	**Weight decay**
No.1	0.5/0.2	AdamW/AdamW	1.049E-3/8.234E-5	32/16	1E-5/1E-2
No.2	0.3/0.5	AdamW/AdamW	1.922E-4/2.852E-3	4/16	1E-3/1E-3
No.3	0.5/0.6	Adagrad/AdamW	9.036E-3/2.362E-2	8/4	1E-4/1E-2
No.4	0.4/0.3	AdamW/AdamW	2.940E-4/2.159E-4	16/8	1E-4/1E-2
No.5	0.4/0.2	AdamW/AdamW	5.330E-5/6.413E-4	4/16	1E-4/1E-4
No.6	0.4/0.4	AdamW/AdamW	2.639E-4/6.915E-4	4/16	1E-4/1E-4
No.7	0.7/0.3	Adagrad/AdamW	1.181E-2/3.533E-4	16/8	1E-2/1E-5
No.8	0.3/0.5	Adagrad/AdamW	2.276E-2/1.058E-3	16/4	1E-5/1E-2
No.9	0.4/0.2	AdamW/AdamW	5.243E-4/2.092E-3	16/16	1E-2/1E-2

### 3.2. Performance of the proposed channel selection method

The proposed method incorporates channel attention learning process and classification during model training through the CA layer. Due to this unique feature, the method can be classified as an embedded technique. To evaluate the channel selection ability of the proposed method, it was compared with two other state-of-the-art embedded techniques: Gumbel-softmax (GS) layer and the automatic channel selection (ACS) layer (Strypsteen and Bertrand, 2021; Zhang et al., 2021).

The network architecture was identical to that of ECA-DeepNet, except for replacing the CA layer with the GS or ACS layer for comparison. The ACS layer is based on another attention module, the SE module (Hu et al., 2018), which has been proven effective and commonly used in many fields (Park et al., 2020; Liu et al., 2021; Zhang and Zhang, 2022). It can be used for channel selection comparison following the steps proposed in this paper. Considering the potential adverse effects between the ECA and SE modules, network with all ECA modules replaced with SE modules was also compared, and it is named All-SE.

**Figure 4:**
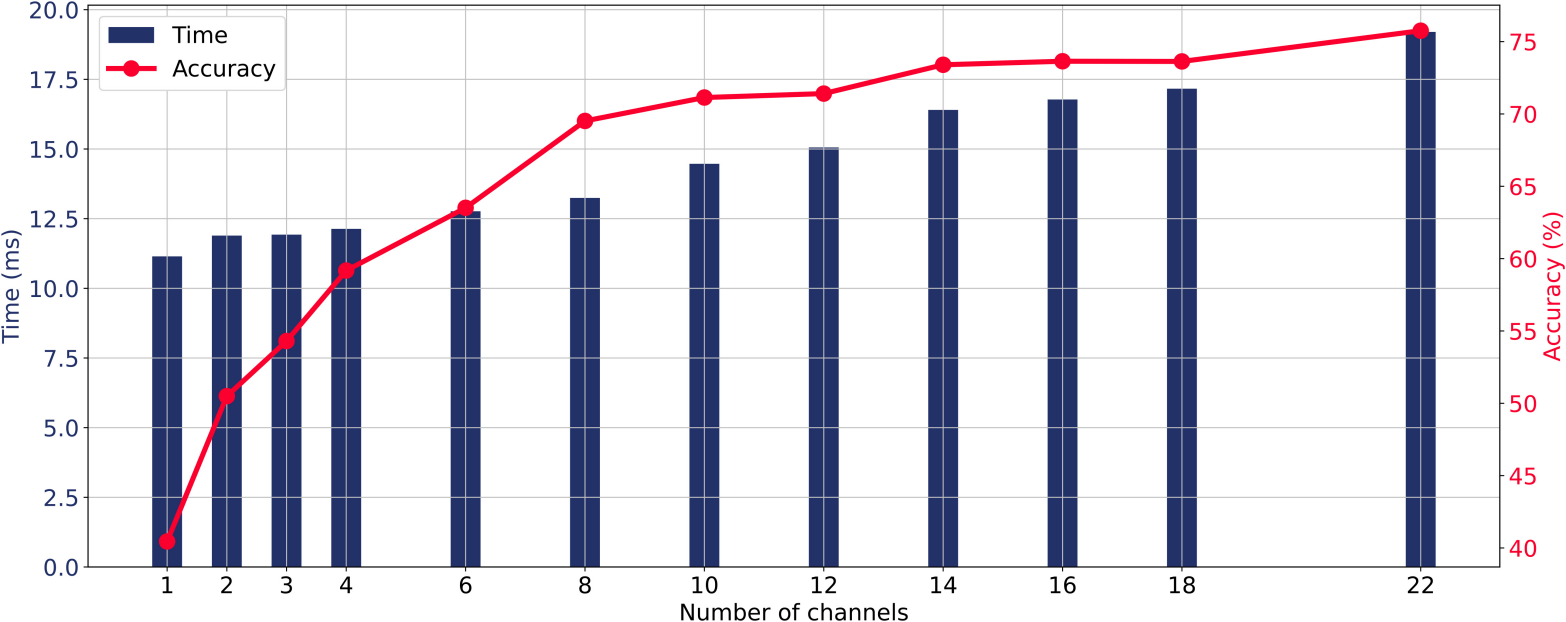
Relationship between the number of input channels, average classification accuracy, and prediction time of the model with the ECA-DeepNet method.

When the CA layer is used for channel selection, the relationship between the number of input channels, average classification accuracy, and prediction time of the model is illustrated in Figure 4. As the number of channels increases, the prediction time and classification accuracy were gradually increasing. The average accuracy curve started to flatten when more than eight channels were involved and the increase in accuracy became slow when more than 14 channels were involved. The aim of this study is to maintain the high accuracy while minimizing the number of input EEG channels. Taking into account the trade-off between real-time requirements and accuracy in MI-BCI, this study takes eight channels as an illustrative case to analyze the channel selection method from all 22 channels and compare different methods.

Table 4 demonstrates the classification accuracy for each subject after selecting eight channels by different channel selection methods. In Table 4, the standard deviation (STD) of accuracy across nine subjects was calculated to evaluate the method's robustness across different subjects. Results show that the CA layer achieved optimal channel selection performance in most subjects, with its average eight-channel accuracy of 69.52%, indicating a 13.81%, 5.46% and 3.17% improvement compared to the GS layer (55.71%), All-SE (64.06%), and the ACS layer (66.35%). The proposed method showed a large improvement in the accuracy of subject No. 4 (up to 7.17%) and No. 7 (up to 12.97%).

**Table 4:** Classification accuracies (%) for eight channels with the GS layer, All-SE, ACS or CA layer.

**Subject**	**Hold-out sccuracy (%)**				**Training set cross-validation accuracy (%)**			

	**GS layer**	**All-SE**	**ACS layer**	**CA layer**	**GS layer**	**All-SE**	**ACS layer**	**CA layer**
No.1	51.27	65.27	**77.89**	76.85	54.79 ± 16.03	69.55 ± 2.85	**71.55** **±4.01**	70.62 ± 4.71
No.2	31.36	47.68	**48.37**	47.10	34.71 ± 9.46	50.92 ± 3.05	52.66 ± 4.80	**53.96** **±6.39**
No.3	81.36	82.63	83.68	**88.54**	71.77 ± 5.76	80.32 ± 4.73	77.98 ± 5.22	**82.52** **±2.71**
No.4	42.36	54.62	55.67	**62.84**	41.66 ± 5.30	52.18 ± 4.87	53.34 ± 5.63	**55.75** **±7.95**
No.5	46.99	57.06	55.67	**61.11**	45.59 ± 2.89	52.99 ± 3.23	52.68 ± 5.51	**59.37** **±1.96**
No.6	36.34	51.27	52.89	**54.62**	38.67 ± 4.70	**53.23** **±3.43**	52.33 ± 2.38	48.96 ± 1.78
No.7	65.39	64.46	69.32	**82.29**	66.43 ± 7.06	64.57 ± 2.81	65.52 ± 1.87	**78.11** **±2.47**
No.8	68.98	73.72	**75.23**	73.26	59.07 ± 6.75	75.23 ± 5.59	75.01 ± 3.59	**78.02** **±5.28**
No.9	77.31	**79.86**	78.47	79.05	68.04 ± 4.83	79.76 ± 4.46	79.64 ± 2.71	**83.13** **±4.23**
Average	55.71	64.06	66.35	**69.52**	53.41 ± 6.97	64.31 ± 3.89	64.52 ± 3.97	**67.82** **±4.17**
STD	18.15	**12.55**	13.23	13.80	13.78	12.34	**11.85**	13.38

The best value in each row is denoted in boldface.

In addition, a 5-fold cross-validation on the training set was also carried out to further assess the robustness and stability of the proposed method. The results of cross-validation are consistent with those of the hold-out method, demonstrating that the CA layer exhibited superior channel selection capabilities compared to other methods.

**Figure 5:**
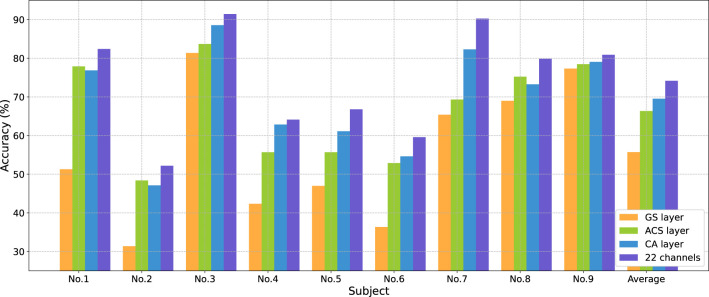
Classification accuracies (%) comparison for each subject in BCIC IV 2a dataset from 22 to eight channels using different channel selection methods.

Figure 5 shows changes in average accuracy before and after using different channel selection methods. The average accuracy decreased by 18.46% (GS layer), and 7.82% (ACS layer) after selecting eight channels from 22 channels. The CA layer performed the best, reducing the number of channels by 63.64% at the cost of a 4.65% decrease in classification performance.

To further evaluate the channel selection capability of the proposed method for different number of channels, its average performance across all subjects was compared with other channel selection methods for different channel subset size. Specifically, comparisons were made for subsets of 10, 12, 14, and 16 channels. These specific channel subset sizes were chosen because accuracy significantly degraded when the number of channels was less than eight, while exceeding 16 channels may impact real-time processing speed, as shown in Figure 4. The hold-out and training set cross-validation results of these comparisons are presented in Table 5. The results indicate that the CA layer consistently demonstrated optimal or near-optimal channel selection capabilities in all scenarios, substantiating the effectiveness and practicality of the proposed method for channel selection in MI-BCI.

**Table 5:** Average classification accuracies (%) across all subjects for 10, 12, 14, and 16 channels with the GS layer, All-SE, ACS or CA layer.

**Number**	**Hold-out accuracy (%)**				**Training set cross-validation accuracy (%)**			

**of channels**	**GS layer**	**All-SE**	**ACS layer**	**CA layer**	**GS layer**	**All-SE**	**ACS layer**	**CA layer**
10	57.24	64.84	69.31	**71.14**	47.74 ± 5.47	65.19 ± 5.21	67.25 ± 4.85	**67.65** **±3.95**
12	54.29	67.81	**72.80**	71.41	46.65 ± 5.82	66.16 ± 4.45	69.67 ± 5.10	**69.93** **±5.57**
14	52.93	69.08	72.22	**73.40**	47.55 ± 4.45	65.78 ± 4.39	69.56 ± 4.31	**71.84** **±4.17**
16	53.43	70.84	73.48	**73.64**	47.03 ± 5.05	67.01 ± 4.29	70.22 ± 5.01	**70.43** **±3.83**

The best value in each row is denoted in boldface.

### 3.3. Position of the selected channels

**Figure 6:**
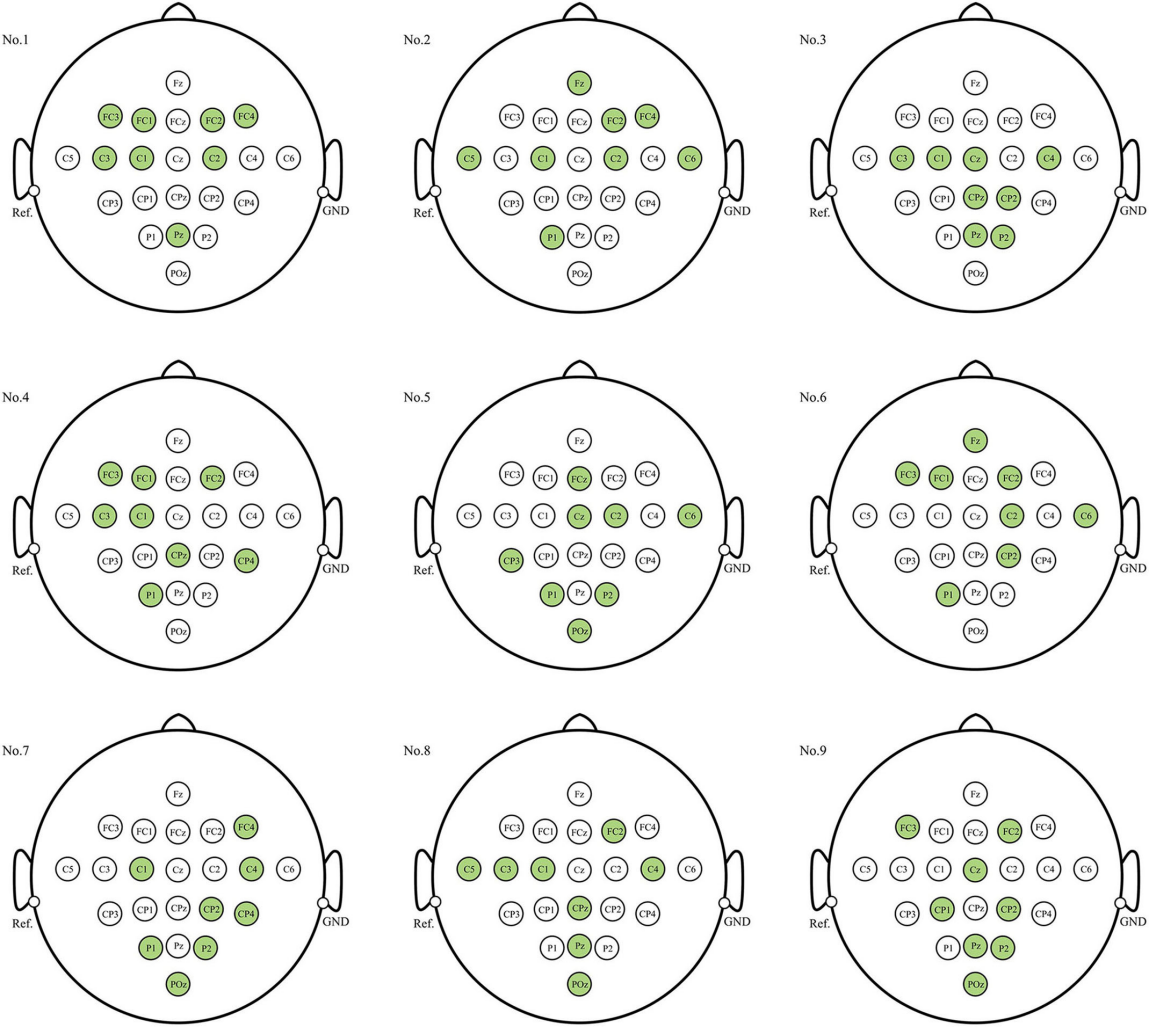
Position of each subject's personalized channel subset in 10–20 system for BCIC IV 2a dataset. The channels marked in green are the selected channels.

When the number of electrodes (or channels) is determined, which channels should be selected is a long-standing issue when decoding EEG signals. Figure 6 illustrates the electrode distribution of the eight-channel subset obtained using the proposed method for each subject on BCIC IV 2a dataset.

The optimal eight-channel subsets varied among each subject. Subjects No.3, No.7, and No.9 exhibited a tendency toward the parietal region (near electrode Pz), responsible for processing somatosensory information, in their eight selected channels. On the other hand, subject No.1 showed a preference for the frontal region (near electrode Fz), associated with motor control and execution, in his eight selected channels. For the remaining subjects, the electrodes were distributed across both parietal and frontal lobe regions. This indicates that both the parietal and frontal regions, in addition to the motor cortex, play crucial roles in the MI process. Furthermore, the eight-channel subset for most subjects were concentrated near the central sulcus region (electrodes C3, C4, Cz), which is considered to have the most evident ERD/ERS phenomena and is most commonly used for MI classification.

### 3.4. Performance of ECA modules

To evaluate the performance of ECA modules on classification accuracy, comparative experiment was taken among EEGNet (Lawhern et al., 2018), which is also a highly-cited model, DeepNet, ECA-DeepNet, and SE-DeepNet. All 22 channels and eight channels selected by proposed method were used for the comparison, and the hold-out and training set cross-validation results are shown in Tables 6, 7.

**Table 6:** Classification accuracy (%) with all 22 channels for EEGNet, DeepNet, SE-DeepNet, ECA-DeepNet methods.

**Subject**	**Hold-out accuracy (%)**					**Training set cross-validation accuracy (%)**				

	**EEGNet**	**DeepNet**	**SE-DeepNet**	**ECA-DeepNet**	**ECA-DeepNet**	**EEGNet**	**DeepNet**	**SE-DeepNet**	**ECA-DeepNet**	**ECA-DeepNet**
					**without CA layer**					**without CA layer**
No.1	75.34	83.45	**86.69**	85.06	82.40	74.09 ± 3.74	77.32 ± 4.14	74.88 ± 4.40	**77.77** **±4.43**	76.29 ± 3.21
No.2	**56.07**	53.82	51.74	54.05	52.9	49.30 ± 5.65	54.61 ± 6.87	53.02 ± 8.53	**59.17** **±6.91**	56.97 ± 7.72
No.3	**91.84**	88.77	84.72	90.85	91.43	**85.66** **±5.47**	82.27 ± 3.50	80.09 ± 3.23	82.97 ± 3.26	82.97 ± 2.02
No.4	61.80	63.08	62.04	**67.93**	64.12	51.99 ± 4.28	54.38 ± 7.80	53.12 ± 7.17	**57.64** **±5.66**	57.05 ± 9.07
No.5	63.71	67.01	67.59	**70.60**	66.78	56.36 ± 5.21	61.10 ± 3.94	63.53 ± 6.60	66.87 ± 5.67	**67.23** **±2.48**
No.6	57.46	57.99	57.29	59.38	**59.60**	53.16 ± 5.33	58.55 ± 4.85	54.02 ± 6.42	**66.80** **±5.37**	59.74 ± 4.06
No.7	81.07	90.16	87.85	**92.36**	90.27	78.82 ± 4.72	**82.98** **±3.79**	79.73 ± 4.12	82.30 ± 2.03	80.00 ± 5.08
No.8	80.55	77.31	76.27	78.47	**79.86**	81.49 ± 3.42	80.21 ± 2.36	81.71 ± 3.81	**81.72** **±1.56**	80.89 ± 3.66
No.9	80.72	**83.45**	79.05	83.10	80.90	**83.81** **±2.40**	81.37 ± 1.89	80.32 ± 1.27	80.11 ± 3.88	82.75 ± 1.17
Average	72.06	73.89	72.58	**75.76**	74.17	68.30 ± 4.47	70.31 ± 4.35	68.94 ± 5.06	**72.82** **±4.31**	71.54 ± 4.27
STD	**12.62**	13.69	13.41	13.60	13.93	15.24	12.73	12.87	**10.24**	11.28

The best value in each row is denoted in boldface.

**Table 7:** Classification accuracy (%) with eight channels for EEGNet, DeepNet, SE-DeepNet, and ECA-DeepNet methods.

**Subject**	**Hold-out accuracy (%)**					**Training set cross-validation accuracy (%)**				

	**EEGNet**	**DeepNet**	**SE-DeepNet**	**ECA-DeepNet**	**ECA-DeepNet**	**EEGNet**	**DeepNet**	**SE-DeepNet**	**ECA-DeepNet**	**ECA-DeepNet**
					**without CA layer**					**without CA layer**
No.1	**77.77**	78.99	73.03	76.85	75.57	71.66 ± 3.74	69.34 ± 3.44	69.70 ± 3.07	70.62 ± 4.71	**72.00** **±2.62**
No.2	47.04	**49.65**	43.28	47.10	45.13	46.33 ± 5.82	50.93 ± 6.99	**54.77** **±6.67**	53.96 ± 6.39	54.28 ± 7.16
No.3	85.41	86.80	87.73	**88.54**	84.60	78.26 ± 3.19	**84.02** **±3.21**	82.88 ± 3.61	82.52 ± 2.71	83.67 ± 2.13
No.4	57.98	**65.63**	57.87	62.84	59.38	**55.89** **±4.33**	49.29 ± 4.51	53.69 ± 6.68	55.75 ± 7.95	53.44 ± 7.67
No.5	50.34	55.03	59.72	**61.11**	59.60	49.08 ± 3.16	55.91 ± 1.96	57.75 ± 3.00	**59.37** **±1.96**	53.67 ± 9.20
No.6	46.88	44.61	51.38	**54.62**	52.89	48.25 ± 3.42	45.14 ± 2.29	45.61 ± 4.73	**48.96** **±1.78**	48.85 ± 3.54
No.7	62.32	61.80	79.39	**82.29**	76.50	64.47 ± 3.63	75.32 ± 3.88	76.38 ± 2.48	78.11 ± 2.47	**81.35** **±3.50**
No.8	60.24	64.93	73.37	73.26	**75.57**	66.19 ± 3.34	76.62 ± 5.09	76.62 ± 4.31	**78.02** **±5.28**	77.68 ± 4.18
No.9	73.95	75.69	77.54	**79.05**	**79.05**	75.93 ± 3.64	81.02 ± 8.28	80.34 ± 4.39	**83.13** **±4.23**	80.68 ± 2.88
Average	62.44	64.79	67.03	**69.52**	67.59	61.78 ± 3.81	65.29 ± 3.58	66.42 ± 4.33	**67.83** **±4.17**	67.29 ± 4.77
STD	13.90	13.90	14.64	13.80	**13.60**	**12.32**	15.00	13.62	13.38	14.41

The best value in each row is denoted in boldface.

It can be found that incorporating ECA modules into the network effectively improved the classification accuracy, and it outperformed the SE module in eight out of nine subjects with both 22 channels and eight channels. The average accuracies of ECA-DeepNet, SE-DeepNet, DeepNet, and EEGNet were 75.76, 72.58, 73.89, and 72.06% respectively with 22 channels and 69.52, 67.03, 64.79, and 62.44%, respectively, with eight channels. The results of cross-validation align with this finding, demonstrating that ECA-DeepNet achieved optimal classification performance in most cases.

The most influential factor leading to the difference between the ECA module and SE module is that the ECA module avoids the side effects of dimensionality reduction on channel attention prediction. The main difference between the two modules is that: To limit model complexity, the SE module adjusts the dimensionality of the two fully connected layers, i.e., by projecting channel features into a low-dimensional space and then mapping them back. At the same time, the ECA module chooses to implement it with 1*D* fast convolution of kernel size *k* or an equivalently dimensioned fully connected layer, maintaining the same channel dimensionality (when used as the CA layer). The dimensionality reduction operation in the SE module destroys the direct correspondence between a channel and its weights, directly leading to SE modules not achieving the same performance as ECA modules.

To evaluate the importance of the CA layer and ECA module in ECA-DeepNet, the performance of ECA-DeepNet without the CA layer was compared to both DeepNet and the complete ECA-DeepNet. The comparison results are shown in Tables 6, 7. It can be observed that removing either the CA layer or the ECA modules between the convolutional layers from ECA-DeepNet resulted in a decrease in accuracy. Removing the CA layer led to a decrease in accuracy by 1.59% with 22 channels and 1.93% with eight channels, while removing all ECA modules resulted in a decrease in accuracy by 1.87% with 22 channels and 4.73% with eight channels. The results of cross-validation align closely with this conclusion. The removal of the ECA module exhibited a more significant impact with eight channels, possibly attributed to these eight channels being selected by the ECA-DeepNet network. The characteristic of the embedded channel selection method being trained alongside the classifier determines that changes in the model structure will result in a misalignment between the selected channels and the new model. This misalignment had a relatively minor effect when only the CA layer was removed, but it introduced a substantial adverse impact when all ECA modules were eliminated.

## 4. Discussion

In this study, a novel embedded channel selection method based on the ECA module is introduced for MI-BCI. The publicly available BCIC IV 2a dataset was employed to compare and evaluate the performance of this method through the hold-out validation and training set cross-validation. The experimental results reveal that, compared to the other two state-of-the-art techniques, the proposed method achieved superior performance in selecting channel subsets (Tables 4, 5). The ECA module, functioning as an attention mechanism, not only serves for channel selection but also contributes to the further enhancement of network performance (Tables 6, 7).

A long-standing challenge pertains to the selection of channels for the decoding of EEG signals when the number of channels is pre-determined. Figure 6 illustrates that even among healthy subjects, the optimal subset of eight channels can exhibit significant variation. While the majority of these channels tended to cluster around the central sulcus, for some individuals, the distribution of eight channels extended to the frontal lobe region, while others showcased distribution within the parietal lobe region, or a combination across both regions. This observation indicates the crucial role played by both the parietal and frontal lobes in the process of MI-EEG decoding. This result is consistent with anatomy and previous studies (Pfurtscheller and da Silva, 1999; Hetu et al., 2013; Park and Chung, 2020), providing the theoretical basis and interpretability to the channel subsets obtained through the proposed method in this paper.

In this study, one of the chosen comparative methods was the SE module (Hu et al., 2018), which is also an channel attention module. The performance of the SE module fell short of the ECA module in most cases (Tables 4–7). This disparity primarily arises from the fact that the SE module introduces dimensionality reduction when forming the bottleneck-like structure. This reduction operation may result in loss of feature information during the process of channel attention learning and disrupt the direct correspondence between channels and attention weights. In contrast, the ECA module usually employs 1*D* convolution operations, allowing each channel to aggregate information from surrounding channels. When it serves as the CA layer, a fully connected layer that does not alter the channel dimension is employed to learn channel attention. These designs enable a more direct capture of interdependencies between channels.

EEG-based MI classification is a prominent research direction. To enhance accuracy, researchers employ an increasing number of electrodes to acquire more comprehensive information. However, different channels contribute to the classification process in distinct ways, and redundant channels may arise. Consequently, an appropriate channel subset through channel selection becomes highly necessary. This paper proposes a novel channel selection method that integrates the ECA module with a CNN. During the training process, the module automatically learns the importance of individual channels based on feature interdependencies and improves performance as well. Based on the extracted channel weights after training, a ranking of channel importance is established. Researchers can select appropriate channel subsets for different subjects based on practical accuracy and hardware requirements from the ranking. Using the BCIC IV 2a dataset, the proposed method was compared with two state-of-the-art embedded channel selection methods, namely ACS Layer and GS Layer. The proposed method achieved an average accuracy of 69.52% with eight channels, outperforming the other two algorithms. The selected eight channels align with prior research and anatomical knowledge. Furthermore, to evaluate the impact of integrating the ECA module into a CNN, a comparison of classification performance was conducted between EEGNet, the original DeepNet, the proposed ECA-DeepNet, and SE-DeepNet. The ECA-DeepNet achieved an accuracy of 75.76% with 22 channels and 69.52% with eight selected channels, exhibiting a 1.93 and 4.73% accuracy improvement over the original DeepNet. The experimental results demonstrate that the ECA module not only assists in channel selection but also improves classification performance. Therefore, this paper introduces a feasible approach for channel selection in EEG-based MI-BCIs.

## Data availability statement

The original contributions presented in the study are included in the article/supplementary material, further inquiries can be directed to the corresponding author.

## Author contributions

LT: Conceptualization, Funding acquisition, Methodology, Validation, Writing—original draft, Writing—review and editing. YQ: Conceptualization, Formal analysis, Methodology, Validation, Writing—original draft, Writing—review and editing. LP: Conceptualization, Formal analysis, Funding acquisition, Methodology, Writing—review and editing. CW: Formal analysis, Validation, Visualization, Writing—original draft. Z-GH: Funding acquisition, Project administration, Supervision, Writing—review and editing.
